# Research on the measurement of China's emergency management capabilities for public health emergencies and their driving factors

**DOI:** 10.3389/fpubh.2026.1785960

**Published:** 2026-08-31

**Authors:** Li Yuan, Xiaocheng Gan, Kexin Zhang, Qianxi Zhang, Xie Yuan

**Affiliations:** Xinjiang University, School of Politics and Public Administration (School of National Security), Ürümqi, China

**Keywords:** dagum gini coefficient, emergency management capabilities, kernel density estimation, public health emergencies, regional disparities

## Abstract

Enhancing regional emergency response capabilities for public health emergencies poses a major challenge for China, which is undergoing rapid urbanization. Based on an analysis of the theoretical framework of emergency management for public health emergencies, this paper constructs an evaluation system comprising four dimensions: prevention and monitoring, emergency response, recovery and reconstruction, and emergency support. It employs the entropy weighting method to measure the emergency management levels of 285 prefecture-level cities in China from 2011 to 2024, utilizes the Dagum Gini coefficient and kernel density estimation to analyze regional disparities and dynamic evolution, and adopts the spatial Durbin model to investigate the spatial effects of driving factors. The study found that the index of emergency management levels for public health emergencies in China shows an overall upward trend but with a slow growth rate, rising from 0.037 during the 12th Five-Year Plan period to 0.048 during the 14th Five-Year Plan period. Spatially, it exhibits a gradient distribution characterized by “higher in the east and lower in the west, higher in the south and lower in the north,” with the eastern and southern regions showing a significant lead. Regional disparities have remained at high levels and continue to widen, with the overall Gini coefficient rising from 0.4836 to 0.5285; inter-regional disparities are the primary source, followed by intra-regional disparities, and the kernel density curve indicates that polarization is deepening. Among the driving factors, digital infrastructure and the level of technological innovation are core positive drivers, both exhibiting significant local promotion effects and positive spatial spillover effects; The overall effect of financial development is significantly negative, primarily due to negative spatial spillovers, reflecting the inhibitory role of cross-regional siphoning of financial resources; competition among local governments constrains neighboring regions through negative spatial spillovers; human capital levels have only a weak positive effect locally, while market openness has no significant impact. Regional heterogeneity is pronounced: the eastern region is driven by both digital infrastructure and technological innovation, with the strongest spillover effects; in the central region, technological innovation and human capital play key roles, while local government competition exhibits negative local effects; in the western region, the driving effect of digital infrastructure is not significant, but financial development has a positive indirect effect; in the northeastern region, digital infrastructure only has a local promotional effect and lacks spillover effects. Strengthening interregional coordination and expanding positive spillover effects are important pathways for improving emergency management capabilities.

## 1 Introduction

Since the beginning of the 21st century, the deepening of globalization has intertwined with China's massive urbanization process. High-density population concentrations, frequent cross-regional mobility, and profound changes in the ecological environment have led to a significant increase in the frequency, transmission speed, and scope of public health emergencies (1). From SARS to the H1N1 influenza pandemic, and from the prevention and control of imported Ebola cases to the COVID-19 pandemic, each major outbreak has clearly demonstrated that public health emergencies are no longer merely medical issues, but rather core non-traditional security concerns that impact national security, economic resilience, and social stability (2). For a vast country like China, with significant disparities in regional development, the rapid expansion of urban clusters has amplified the potential for the spread of public health risks while boosting economic efficiency. Enhancing the level of emergency management for public health emergencies is not only a fundamental requirement to safeguard the lives and health of the people but also a necessary prerequisite for ensuring the sustained advancement of Chinese-style modernization (3, 4). Over the past decade, China has elevated the development of its public health emergency response system to an unprecedented strategic level. From the “Healthy China 2030” Plan Outline to the Law on the Response to Public Health Emergencies, China has established a relatively comprehensive institutional framework (5), driving the transformation of emergency capacity building from reactive measures to full-cycle risk management (6). However, despite intensive top-level planning and sustained increases in fiscal investment, the actual performance of China's public health emergency governance exhibits a complex duality. On the one hand, overall national capacity has indeed improved (7). The direct reporting system of the disease control network now covers 84,000 healthcare institutions, and major epidemic treatment bases and emergency medical rescue networks are gradually taking shape; on the other hand, due to deep-seated constraints such as regional economic development gradients, spatial mismatches in medical resources, disparities in fiscal self-sufficiency, and heterogeneity in institutional environments, the interprovincial gap and regional divergence in emergency response capabilities have not automatically narrowed with increased investment (8). Developed citys in the eastern and central regions, leveraging stronger existing medical resources, more robust digital infrastructure, and more efficient financial service systems, are able to rapidly achieve a closed-loop monitoring–response–treatment system; in contrast, western and some northern regions, constrained by tight fiscal constraints, brain drain, and insufficient market openness, face path lock-in in capacity accumulation (9). More notably, under the current administrative performance evaluation framework, competition among local governments may lead to the relative marginalization of public goods with long-term, implicit returns such as public health, while cross-administrative boundary joint prevention and control efforts become fragmented due to the logic of territorial management. Consequently, the core issues facing China today have shifted to whether development outcomes are equitably accessible, whether regional disparities are widening, and which factors are substantively driving or hindering capacity enhancement (10, 11).

Based on this, this paper uses more than 200 prefecture-level cities in China as observation units to construct an evaluation system for emergency management levels covering four dimensions: prevention and monitoring, emergency response, recovery and reconstruction, and emergency support. It employs the entropy weighting method to measure the dynamic evolution of emergency management levels in these cities from the 12th Five-Year Plan period to the 14th Five-Year Plan period, utilizing the Dagum Gini coefficient and kernel density estimation to reveal the structural origins and distributional patterns of regional disparities over time. We then empirically identify the driving directions and regional heterogeneity of key factors such as digital infrastructure, financial services, local government competition, market openness, and human capital, aiming to provide a policy basis for enhancing the national emergency management level for public health emergencies.

The marginal contributions of this paper are as follows: (1) We have established a four-dimensional evaluation framework comprising prevention and monitoring—emergency response—recovery and reconstruction—emergency support, incorporating digital infrastructure and financial safeguards into the institutional support indicators to achieve an integrated assessment of the entire process and all elements of emergency management; (2) Based on panel data from more than 200 prefecture-level cities spanning the 12th to the 14th Five-Year Plans, this study employs a combined tool of the Dagum Gini coefficient and kernel density estimation to systematically uncover, for the first time, the structural origins and distributional evolution of regional disparities in China's emergency management capabilities for public health emergencies; (3) By incorporating digital infrastructure, financial services, local government competition, market openness, and human capital into a unified econometric model, and using subregional regression to reveal the heterogeneous pathways of driving mechanisms, this study deepens the theoretical understanding and policy implications regarding regional disparities in emergency management capabilities.

**Figure 1:**
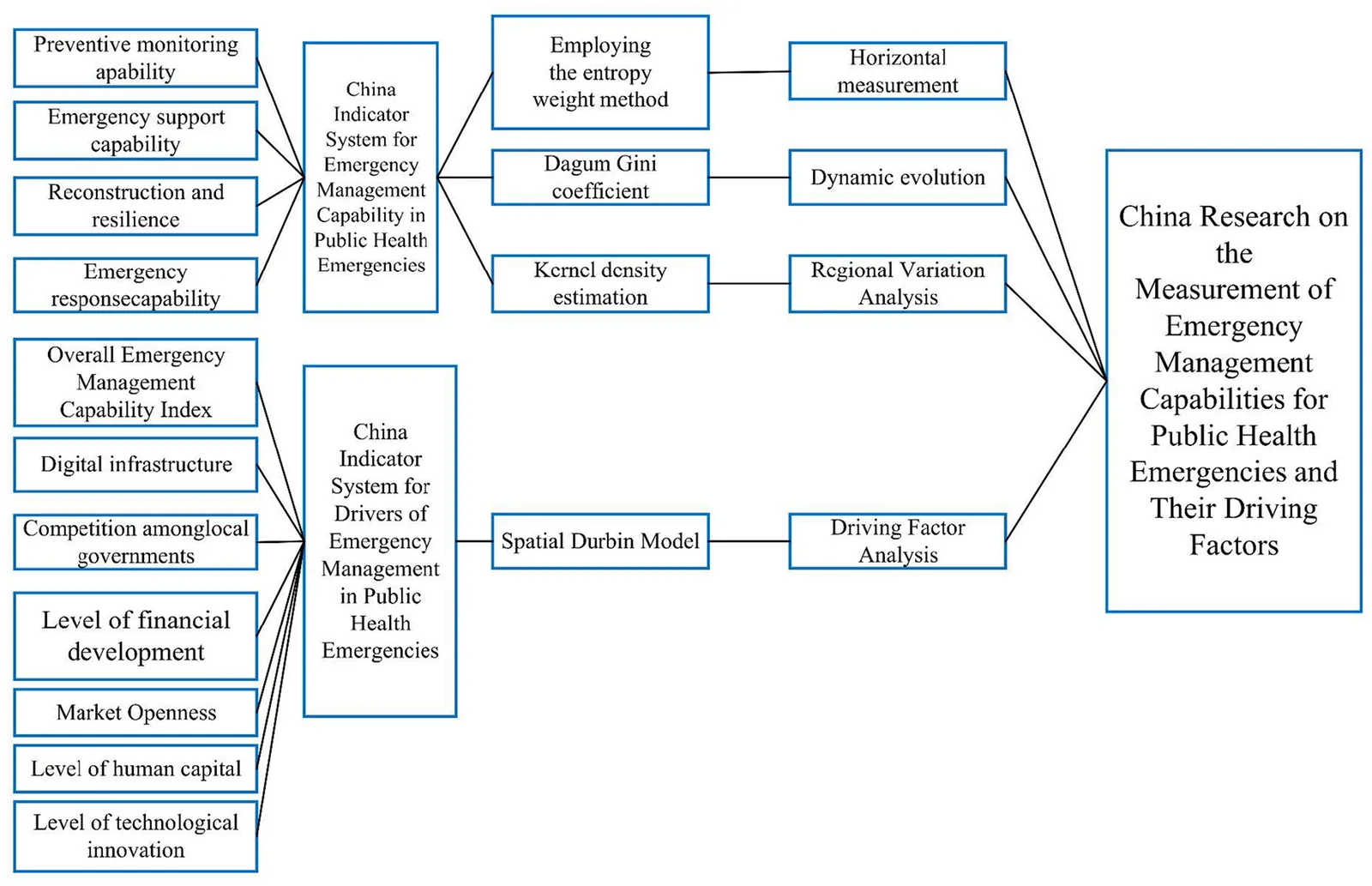
Technology roadmap.

The structure of this paper is as follows: Part Two presents a literature review on emergency management for public health emergencies. Part Three introduces four models—entropy weighting, the Dagum Gini coefficient, kernel density estimation, and the spatial Durbin model—as well as the sources of the indicator data. Section 4: First, it measures the level of emergency management for public health emergencies in China using entropy weighting; second, it employs the Dagum Gini coefficient and kernel density estimation to analyze dynamic evolution and regional differences; third, it uses the spatial Durbin model to examine whether the driving factors significantly influence emergency management for public health emergencies. Section 5 presents the conclusions and implications. The research roadmap is shown in Figure 1.

## 2 Literature review

Academic research on emergency management for public health emergencies can be broadly categorized into three areas: first, research on the construction of emergency management indicator systems, which focuses on how to scientifically design evaluation frameworks that comprehensively reflect emergency management capabilities; second, research on the quantitative measurement of emergency management levels and regional disparities, which examines the spatial variation and dynamic evolution of these capabilities; and third, research on driving factors and mechanisms, which aims to identify the institutional, technological, and behavioral variables that influence emergency management levels.

### 2.1 Research on the construction of emergency management indicator systems

Regarding how to construct an evaluation system that comprehensively reflects the entire emergency management process, two representative approaches have emerged in the academic community. The first follows a “full life cycle” logic, dividing the emergency management process into stages such as prevention, preparedness, response, and recovery, and establishing indicators accordingly. Nikseresht et al. (12) used this framework to construct a matrix for the full-process emergency management of public health emergencies in higher education institutions, providing a logical prototype for the phased classification of indicators. The advantage of this approach lies in its alignment with the temporal characteristics of emergency management, allowing for the establishment of targeted indicators at each stage and avoiding excessive bias in evaluation results toward any single phase. The second approach adopts a “resource endowment—capacity output” logic, emphasizing the comparative relationship between input conditions and output performance. Hu et al. (13) constructed an evaluation system based on four dimensions—multi-stakeholder coordination, healthcare security, stable socioeconomic operations, and infrastructure development—and used the entropy method to assess panel data from 31 citys for the period 2013–2019. Zhu et al. (14) focused on rural settings, refining 29 indicators across four dimensions, and used factor analysis and cluster analysis to reveal a spatial gradient where the southeastern region performed best, followed by the central region, with the northwestern region lagging behind. Khan et al. (15), however, shifted their focus to the community level. Drawing on identity theory, they constructed an evaluation framework based on four sources of capability, including political identity and competence identity. Their empirical findings indicate significant differences among communities in soft capabilities such as competence and emotional identity, highlighting that evaluation systems must not focus solely on existing hardware while neglecting the institutional support dimension.

The aforementioned studies provide important insights for this paper but also reveal two shortcomings: First, most indicator systems emphasize the stock of medical resources and post-event treatment capabilities, while paying insufficient attention to forward-looking indicators for prevention and monitoring, as well as institutional indicators such as digital infrastructure and financial safeguards involved in recovery and reconstruction and emergency support; Second, while the “full life cycle” framework is theoretically sound, in practice it often simplifies the “emergency support” dimension—which runs throughout the entire process—by embedding it into a specific stage rather than presenting it as an independent component. Building upon the strengths of the life-cycle framework's phased approach and the resource endowment framework's multidimensional coverage, this paper constructs a four-dimensional evaluation system comprising “prevention and monitoring—emergency response—recovery and reconstruction—emergency support.” The first three dimensions correspond to the sequential stages of emergency management, while the “emergency support” dimension independently delineates the supporting conditions—such as information infrastructure, financial safeguards, and human resource reserves—that span the entire process, thereby achieving “full-functional integration.”

### 2.2 Research on quantitative measurement of emergency management capabilities and regional disparities

In terms of evaluation methods, early studies largely relied on subjective weighting approaches such as the Analytic Hierarchy Process (AHP) and the Delphi method. In recent years, objective weighting methods such as Coupled Coordination (16), entropy-weighted TOPSIS (17, 18), factor analysis (19), and Data Envelopment Analysis (DEA) have gradually become mainstream (20–22). An et al. (19) systematically reviewed the evolution of domestic and international assessment tools, highlighting the Joint External Evaluation (JEE) tool and calling for the establishment of an evaluation system that integrates international standards with local adaptability; Lancaster et al. (23) proposed a chain-based capacity-building approach centered on risk early warning, emergency medical reserves, and talent reserves from a policy implementation perspective. Regarding the identification of regional disparities, Ramsbottom et al. (24) found that China's overall emergency response capacity exhibits polarization and a “long-tail effect,” with the impact of emergency response capacity on prevention and control quality showing significant regional heterogeneity; Han and Peng (25) further pointed out that the gap on either side of the Hu Huanyong Line has deep structural roots, and shortcomings in a single dimension can limit the overall level through the bucket effect.

However, existing quantitative studies still face significant limitations in terms of temporal scope and analytical depth: First, there is a lack of long-term dynamic tracking. Most evaluations focus on a single year or a short time window, failing to cover the complete policy cycle from the 12th Five-Year Plan to the 14th Five-Year Plan—a period marked by major institutional reforms and the impact of the pandemic—and thus struggle to capture trend-based inflection points and phased characteristics in capability evolution. Second, the decomposition of regional disparities lacks sufficient granularity. Existing studies often remain at the level of mean comparisons or simple coefficients of variation, with very few employing the Dagum Gini coefficient to systematically decompose overall disparities into three major sources: intra-regional, inter-regional, and super-variation density. Consequently, they cannot precisely identify which pair of regional groups' disparities is driving the overall imbalance. Third, there is a lack of dynamic characterization of distribution patterns. Kernel density estimation, an effective tool for revealing trends of distribution polarization, convergence, or divergence, has not yet been fully utilized in the field of emergency management for public health emergencies. This paper adopts the entropy-weighted method as a measurement tool, which not only avoids subjective weighting biases but also accommodates the need for longitudinal comparisons of multi-year panel data. Furthermore, by combining the Dagum Gini coefficient with kernel density estimation—the former revealing the structural sources of disparity and the latter depicting the dynamic evolution of distribution patterns—the two approaches complement each other to form a multidimensional analysis of regional disparities.

### 2.3 Research on driving factors and mechanisms

Regarding the driving factors influencing the level of emergency management, the academic explanatory framework has increasingly shifted from a single-variable approach to a composite perspective that intertwines institutional incentives, technological embeddedness, and actor behavior.

At the institutional and behavioral levels, Xue et al. (26) and Shen and Zhang (27), through computational analysis of central and local policy texts, found that excessive rigidity in local governments' territorial management gives rise to an inherent motivation to avoid responsibility. This manifests as two risk-avoidance strategies—procedural compliance and escalation at each level—leading to delayed decision-making and inadequate risk-sharing. This finding suggests that competition among local governments and the performance evaluation system may crowd out resources for public goods with long-term, implicit benefits—such as public health—constituting a deep-rooted institutional cause of regional disparities in emergency response capabilities. Scholars such as Burkle Jr (28), Sun et al. (29), and Nelson et al. (30) have identified core challenges in the public health sector at the level of the legal environment, including the absence of overarching legislation, poor coordination of legal norms, and a weak emergency legal oversight system, confirming that institutional fragmentation and gaps in legal foundations directly constrain operational capabilities. Ipe et al. (31) analyzed selection biases in emergency management policy tools from the perspective of instrumental rationality, calling for the optimization of the tool structure to avoid reliance on a single approach. At the level of technology empowerment, Yazdi et al. (32) proposed a smart public health emergency management framework, elevating digital technology to the foundational support of holistic governance and emphasizing the critical role of smart, multi-point trigger monitoring and early warning systems, as well as multi-departmental data coordination; (11) began with sentiment analysis of social media sentiment to examine spatial and temporal variations in public evaluations of community emergency management, demonstrating that digital information flows have begun to enter the assessment and feedback chain of emergency management. These studies collectively point to a conclusion: digital infrastructure is becoming the core lever for a leap in emergency management capabilities. At the level of economic and social conditions, existing research generally confirms the positive role of traditional variables such as fiscal investment, urbanization rates, and human capital. However, there is insufficient attention to the role of financial service efficiency in ensuring emergency material reserves and post-disaster recovery, and there is a lack of systematic examination of how market openness indirectly enhances emergency response capabilities through resource allocation efficiency (54–57).

Overall, in international comparisons and theoretical frameworks, emergency management research in developed countries places greater emphasis on social capital, community resilience, and public-private partnerships, whereas Chinese academia focuses more on the interactions among central-local relations, fiscal incentives, and technological embedding. The negative spatial spillovers of local government competition identified in this study align with the race to the bottom prediction in fiscal competition theory; the positive spillover effects of digital infrastructure resonate with the proposition in state-capacity theory that infrastructure power enhances governance effectiveness. Unlike Western countries, which rely on market-based mechanisms, the enhancement of China's emergency management capabilities depends more on government-led technology diffusion and cross-regional administrative coordination. This finding enriches the application of developmental state theory in the field of public safety governance.

Furthermore, existing studies on driving factors have two shortcomings: First, the set of variables urgently needs updating. Although digital infrastructure and financial services—as new factors of production—have been frequently cited in normative studies, they have not yet been systematically empirically tested as independent explanatory variables in city-level long-panels. Second, there is insufficient evidence regarding heterogeneity. There is a lack of evidence from a single analytical framework to determine whether the direction and intensity of the aforementioned factors differ significantly across eastern, central, and western regions, or whether the inhibitory effect of local government competition exhibits non-linear characteristics under different fiscal endowment conditions. This paper incorporates five core variables—digital infrastructure, financial services, local government competition, market openness, and human capital—into its analysis of driving factors. By conducting region-specific regression tests to examine their heterogeneous effects, the study aims to challenge the homogeneity assumption of existing research—which treats the entire nation as a single entity—and reveal the differentiated pathways of driving mechanisms under varying regional endowment conditions.

In summary, while existing research has laid a solid foundation for this study, there remains room for further exploration in three areas. First, the evaluation framework is insufficient. Most indicator systems place excessive emphasis on existing medical resources and post-incident treatment, while forward-looking indicators for prevention and monitoring, as well as cross-cutting institutional indicators such as digital infrastructure and financial safeguards, are often oversimplified. Consequently, the measurement results reflect “healthcare capacity” rather than comprehensive emergency management capabilities. Second, the dynamics of evolution and the sources of disparities have long remained a black box. There is a lack of continuous tracking covering the full policy cycle from the 12th Five-Year Plan to the 14th Five-Year Plan, as well as a lack of detailed analysis of the structural causes of regional imbalances using the Dagum Gini coefficient and kernel density estimation. Third, the set of variables representing driving factors and tests of heterogeneity are insufficient. The positive driving role of digital infrastructure and financial services has not been firmly established in the prefecture- and city-level panel data, and the potential crowding-out effects of local government competition and their interaction with regional endowments have not been sufficiently examined.

## 3 Indicator system, data sources and research methodology

### 3.1 Indicator system

#### 3.1.1 Measurement of China's emergency management capabilities for public health emergencies

Emergency management constitutes a complex systemic assessment involving multiple factors and numerous potential indicators. However, when establishing an evaluation framework for the emergency management level of public health emergencies, principles of comprehensiveness, systematicity, scientific rigor, representativeness, and feasibility must be adhered to. This paper draws upon relevant indicators from the Chinese National Emergency Response Plan for Public Health Emergencies, while also incorporating research from scholars [e.g., (33–37)]. Building upon existing research, Combining the PPRR model, which is widely used internationally, with the Law of the People's Republic of China on Responding to Emergencies and the National General Emergency Plan for Public Emergencies issued by the Chinese government, the existing indicators were extensively expanded and optimized. Considering the characteristics of public health emergencies, an indicator system for China's public health emergency management level was constructed. Preventive monitoring capability, emergency handling capability, reconstruction and recovery capability, and emergency response capability were selected as secondary indicators. Subsequently, 16 tertiary indicators were extracted from these four secondary indicators.

This indicator system uses the PPRR model as its framework to assess the level of emergency management for public health emergencies across four dimensions: preventive monitoring, emergency response, reconstruction and recovery, and emergency preparedness. Regarding preventive monitoring capabilities, the number of participants in the Basic Medical Insurance for Urban and Rural Residents, combined with the number of participants in the Basic Medical Insurance for Urban Employees, collectively weaves a dense safety net for medical security. This serves as a positive measure of the baseline level of social protection, ensuring that people can seek timely medical care in emergencies and facilitating the early detection of cases; the two systems are highly complementary. Public health technology expenditure reflects a region's investment in scientific prevention measures such as testing and early warning systems, serving as the technical foundation for enhancing monitoring sensitivity; education expenditure lays the social cognitive foundation for risk communication by influencing public health literacy and compliance with protective measures; meanwhile, the number of recipients of urban and rural social assistance highlights society's inherent vulnerability from the opposite perspective—the larger the population receiving minimum living allowances, the greater the difficulty in implementing early protection and monitoring and early warning. Regarding emergency response capacity, the number of hospitals directly determines the physical capacity for centralized treatment, while healthcare professionals constitute the core treatment force; the number of public transportation vehicles per 10,000 people is innovatively used as a proxy for logistical support potential, reflecting the reserve capacity for personnel transport and material distribution during emergencies; Urban population density serves as the risk baseline, measuring the transmission risks and resource pressures faced during response operations. Regarding reconstruction and recovery capabilities, the number of hospital beds in healthcare institutions provides the capacity foundation for post-event rehabilitation and long-term health management; local fiscal revenue captures the economic lifeline of recovery—the “money bag”—while local government expenditures on social security and employment precisely target “job stability,” serving as the key to resilient recovery by safeguarding people's livelihoods and preventing pandemic-induced poverty. Regarding emergency response capabilities, public health R&D expenditure reflects the potential for scientific and technological breakthroughs during crises and represents the dynamic cash flow for the rapid development of diagnostic kits and vaccines; total expenditure on healthcare institutions serves as an indicator of operational scale, reflecting the depth of the healthcare system's pool of activatable and deployable resources; and the number of healthcare institutions indicates the coverage density of the grassroots network, directly determining the response speed of grid-based screening and community health monitoring.

Regarding the diagnosis of indicator correlation, the variance inflation factor (VIF) was calculated to test for multicollinearity among the various third-level indicators. The results showed that the VIF values for all indicators were below 5 (mean 2.31), with the highest value being VIF = 4.82 between “Number of Beds in Healthcare Institutions” and “Healthcare Professionals.” Since this did not exceed the common threshold of 10, it indicates that there are no serious multicollinearity issues. The Pearson correlation matrix revealed that the correlation coefficient between “number of participants in the basic medical insurance for urban and rural residents” and “number of participants in the basic medical insurance for urban employees” was 0.78, indicating a strong correlation within a reasonable range, reflecting the complementary nature of the two medical insurance systems. The correlation coefficients among the remaining indicators were all below 0.7. Therefore, the indicators selected in this study possess good discriminatory power and independence. The specific indicators selected are shown in Table 1.

**Table 1:** Indicator system for evaluating China's emergency management capabilities for public health emergencies.

Target layer	Policy layer	Metrics layer
China's emergency management capability for public health emergencies	Preventive monitoring capability (A)	A1 Number of participants in the basic medical insurance scheme for urban and rural residents
		A2 Public expenditure on public health technology (proportion of public finance investment in science and technology)
		A3 Number of participants in the basic medical insurance scheme for urban employees
		A4 Education expenditure
		A5 Number of recipients of social assistance in urban and rural areas of China (number of people receiving subsistence allowances)
Emergency management level for public health emergencies	Emergency support capability (B)	B1 Number of hospitals
		B3 Number of public transport vehicles per 10,000 people
		B4 Urban population density
		B5 Healthcare technicians
	Reconstruction and resilience (C)	C1 Number of beds in healthcare institutions
		C2 Local government revenue
		C3 Local Government Expenditure on Social Security and Employment (Data on Social Security and Employment Expenditure from General Public Budgets of Chinese Prefecture-Level Cities)
	Emergency response capability (D)	D1 Public health research and development expenditure
		D2 Total expenditure of healthcare institutions (the aggregate of all costs incurred by all healthcare institutions in the region over a specified period in providing healthcare services)
		D3 Number of healthcare facilities

#### 3.1.2 Driving to in China's emergency management of public health emergencies

When selecting factors relevant to emergency management of public health incidents in the region, two points must be considered: firstly, the chosen factors should be those commonly employed in existing research; secondly, they must not duplicate indicators within the public health emergency management capability framework. Following a systematic review of relevant literature, the following factors were selected, Digital infrastructure (38, 39); Local government competition (40, 41); Financial development level (42, 43); Market openness (44, 45); Human capital levels (46, 47), as drivers of China's emergency management for public health emergencies. This study focuses on six theory-driven core drivers. The model incorporates spatial and time fixed effects to account for regional heterogeneity and common temporal trends. Since the dual-fixed-effects specification of the spatial Durbin model already controls for a large number of potential confounding factors, this study does not include additional macro-level control variables to avoid multicollinearity and overparameterization. As shown in Table 2.

**Table 2:** Framework of driving factors for emergency management of public health emergencies in China.

Variable	Explanation
Dependent variable	
Overall Emergency Management Capability Index	National emergency management level index for public health emergencies as measured by the entropy-based method
Explanatory variable	
Digital infrastructure	Number of internet broadband access users/Year-end resident population (number/person)
Competition among local governments	(Highest per capita GDP of neighboring city/Per capita GDP of this city) × (Highest per capita GDP nationwide/Per capita GDP of this city)
Level of financial development	The ratio of outstanding loans in various categories to local gross domestic product across different regions
Market openness	Foreign Direct Investment (FDI) as a proportion of regional GDP
Level of human capital	Ratio of higher education enrolment to year-end population
Level of technological innovation	Number of regional invention patents granted

### 3.2 Data source

Taking into account data availability and continuity, this study employs 31 citys across China from 2011 to 2023 as its research sample. Data sources include the China Statistical Yearbook, China Regional Economic Yearbook, China Health Statistics Yearbook, and provincial yearbooks.

### 3.3 Research methodology

#### 3.3.1 Entropy rights method

Entropy values reflect the utility value of indicator information, serving as a measure of uncertainty in random events. The greater the randomness of an event, the higher its corresponding entropy value; conversely, the greater the certainty of an event—to the point of inevitability—the lower its entropy value, eventually reaching zero (48). The entropy weighting method assumes that the lower the information redundancy and the greater the variability between indicators, the higher the weight. Its core limitations lie in: (1) the assumption of indicator independence—if there is strong correlation among indicators, entropy weighting will underestimate the importance of overlapping information; (2) sample dependency—weights change as samples are updated, so cross-period comparisons must be made with caution. To mitigate this issue, this paper employs a method of independent weighting across periods followed by averaging, ensuring that the weights reflect the relative importance of each stage while avoiding time-varying biases associated with fixed weights. The steps for determining indicator weight coefficients using the entropy method are as follows:

**Step 1: Data standardization**.

Assume there are *m* evaluation objects (prefecture-level cities) and *n* evaluation indicators. The raw data matrix is **X** = (_*x*_*ij*_)*m*×*n*_. To eliminate the effects of different units and magnitudes, the positive indicators (larger-is-better) and negative indicators (smaller-is-better) are standardized separately. All indicators selected in this study are positive, and the min-max normalization is used:


$$\begin{array}{l} y_{ij}=\frac{x_{ij}-\min\left(x_{j}\right)}{\max\left(x_{j}\right)-\min\left(x_{j}\right)} \end{array}$$ (1)

Equation 1 where *x*_*ij*_ is the original value of the *i*-th object on the *j*-th indicator, min(*x*_*j*_) and max(*x*_*j*_) are the minimum and maximum values of the *j*-th indicator across all objects, and *y*_*ij*_ is the standardized value, with 0 ≤ *y*_*ij*_ ≤ 1. To avoid zero values when logarithms are taken in later steps, the standardized data are shifted by 10^− 5^.

**Step 2:** **Equation 2** **calculate the proportion of the** *i***-th object on the** *j***-th indicator**.


$$\begin{array}{l} p_{ij}=\frac{y_{ij}}{\sum_{i=1}^{m}y_{ij}},i=1,\ldots ,m;\;j=1,\ldots ,n \end{array}$$ (2)

**Step 3:** **Equation 3** **Calculate the information entropy of the** *j***-th indicator**.


$$\begin{array}{l} e_{j}=-\frac{1}{\ln\;m}\overset{m}{\underset{i=1}{\sum }}p_{ij}\ln\left(p_{ij}\right),j=1,\ldots ,n \end{array}$$ (3)

The constant $\frac{1}{\ln\;m}$ ensures that 0 ≤ *e*_*j*_ ≤ 1. If *p*_*ij*_ = 0, we define *p*_*ij*_ln(*p*_*ij*_) = 0.

**Step 4:** **Equation 4** **calculate the information utility value (coefficient of variation) of the** *j***-th indicator**.


$$\begin{array}{l} d_{j}=1-e_{j} \end{array}$$ (4)

A larger utility value indicates a stronger discriminating power of the indicator and thus a higher importance in the comprehensive evaluation.

**Step 5:** **Equation 5** **determine the entropy weight of the** *j***-th indicator**.


$$\begin{array}{l} w_{j}=\frac{d_{j}}{\sum_{k=1}^{n}d_{k}},j=1,\ldots ,n \end{array}$$ (5)

Obviously,$\overset{n}{\underset{j=1}{\sum }}w_{j}=\;1$.

**Step 6:** **Equation 6** **calculate the comprehensive score of each evaluation object**.


$$\begin{array}{l} S_{i}=\overset{n}{\underset{j=1}{\sum }}w_{j}\cdot y_{ij} \end{array}$$ (6)

Here*S*_*i*_is the total index of public health emergency management capacity for the *i*-th city in the corresponding year.

Because this study spans multiple years (2011–2024) and the importance of indicators may change over time, we adopt a period-wise independent weighting approach. Specifically, the above steps are performed separately for three phases: the 12th Five-Year Plan period (2011–2015), the 13th Five-Year Plan period (2016–2020), and the 14th Five-Year Plan period (2021–2024). The scores from the three phases are then concatenated to form the complete panel dataset. This approach captures the dynamic evolution of indicator importance across different stages and is more reasonable than using a single fixed weight for the whole sample.

#### 3.3.2 Dagum gini coefficient method

This paper employs the Dagum Gini decomposition method to analyse regional disparities in China's emergency management of public health incidents, exploring the underlying causes of these differences. Unlike regional disparity quantification methods such as the Theil index, which overlook the specific distribution patterns within sub-samples, the Dagum Gini method accurately reflects both the magnitude and sources of relative disparities (49). Employing this methodology, the overall Gini coefficient decomposes sequentially into contributions from intra-regional variation, net inter-regional differences, and excess variation density. Generally, a lower overall Gini coefficient indicates reduced regional disparity, while a higher coefficient signifies weaker inter-regional coordination (50).

#### 3.3.3 Kernel density estimation method

Kernel density estimation, as a quantitative tool, is widely applied in evaluating regional imbalances in development, revealing changes in absolute regional disparities through continuous density curves. This paper comprehensively examines kernel density curves across four dimensions: distribution location, morphology, extensibility, and polarization trends (51). Equation 7 represents the density distribution function for the emergency management index of public health emergencies: The density distribution function for the emergency management index of public health emergencies is given by Equation 7:


$$\begin{array}{l} \begin{array}{c} f\left(x\right)=\frac{1}{Nh}\sum_{i=1}^{N}K\left(\frac{X_{i}-x}{h}\right)\\ K\left(x\right)=\frac{1}{\sqrt{2\pi }}\exp\left(-\frac{x^{2}}{2}\right) \end{array} \end{array}$$ (7)

Here, N denotes the number of citys within each region; Xi represents the independently and identically distributed observation, i.e., the emergency management index for public health emergencies in each city; x denotes the mean value of the emergency management index for public health emergencies within the region; K denotes the kernel density; h denotes the bandwidth. A smaller bandwidth yields higher estimation accuracy for the kernel density but lower curve smoothness, whereas a larger bandwidth produces a smoother curve but lower estimation accuracy. For the bandwidth component, Silverman's empirical rules were applied to determine the optimal bandwidth for kernel density estimation in each region, based on sample size and data distribution characteristics. This approach ensures that the estimated results accurately reflect the distribution dynamics within each area.

#### 3.3.4 Spatial dubein model

Among various spatpial econometric models, the spatial Durbin model stands as a widely recognized framework capable of simultaneously capturing spatial dependencies in both dependent and independent variables, offering the most flexible and robust spatial effect decomposition framework. By adjusting its coefficients, the spatial Durbin model can be transformed into more commonly used models such as the spatial lag model (SLM) and spatial error model (SEM), rendering it a versatile tool for empirical analysis (52). Consequently, this study proposes employing the following spatial Durbin model for empirical research, analyzing the direct and indirect impacts of relevant influencing factors on emergency management of public health incidents across China's 285 cities.

Spatial lag models (SLMs), spatial error models (SEMs), and spatial Durbin models (SDMs) constitute the three principal components of spatial econometric models. Compared to other spatial econometric models, the spatial Durbin model represents a standardized structure capable of reflecting various spatial spillover effects. Under different coefficient specifications, it can be transformed into common spatial lag models and spatial error models, rendering it more suitable for general application (53). Consequently, this study developed a Spatial Durbin Model incorporating both temporal and spatial effects for empirical testing:


$$\begin{array}{l} B_{it}=\alpha +\rho \overset{n}{\underset{j=1}{\sum }}W_{ij}^{de}B_{jt}+\beta_{0}A_{it}+\beta_{1}Y_{it}+\theta_{0}\overset{n}{\underset{j=1}{\sum }}W_{ij}^{de}A_{it}+\theta_{1} \end{array}$$ (8)

$$\begin{array}{l} \overset{n}{\underset{j=1}{\sum }}W_{ij}^{de}Y_{it}+\mu_{i}+\varphi_{i}+\;\varepsilon_{it} \end{array}$$

Equation 8, i denotes the year, t denotes the city, α denotes the constant term, ρ and θi denote the spatial lag regression coefficients,βi denotes the regression coefficient,μi and σi denote the spatial fixed effects and time fixed effects, respectively,ε(it) denotes the random disturbance term, Y denotes the control variables, and W(ij)d denotes the geographically economic nested spatial weighting matrix.

This study employs a geo-economic nested spatial weight matrix *W*^*de*^ that simultaneously reflects geographical proximity and economic similarity between cities. The construction involves three steps.

**Step 1: Geographical distance weight matrix** **W**^**geo**^

We use the inverse of the spherical distance between cities as the geographical weight. Let the geographic coordinates of city *i* and city *j* be (*lon*_*i*_, *lat*_*i*_) and (*lon*_*j*_, *lat*_*j*_), respectively. The spherical distance *d*_*ij*_ is calculated using the Haversine formula:


$$w_{ij}^{geo}=\{\begin{array}{cc} 1/d_{ij},\; & i\neq j\\ 0,\; & i=j \end{array}$$

Where *R* = 6, 371 km is the Earth's mean radius, and latitudes and longitudes are converted to radians. The geographical weight matrix is defined as the inverse distance:


$$w_{ij}^{econ}=\{\begin{array}{cc} 1/|pgdp_{i}-pgdp_{j}|,\; & i\neq j\\ 0,\; & i=j \end{array}$$

**Step 2: Economic distance weight matrix** **W**^**econ**^

We use the inverse of the absolute difference in per capita GDP to reflect economic similarity. Let *pgdp*_*i*_ and *pgdp*_*j*_ be the average per capita GDP of city *i* and city *j* over the sample period (to smooth out annual fluctuations). The economic weight matrix is defined as:


$$w_{ij}^{econ}=\{\begin{array}{cc} 1/|pgdp_{i}-pgdp_{j}|,\; & i\neq j\\ 0,\; & i=j \end{array}$$

**Step 3: Nested construction**

Both matrices are first row-standardized so that each row sums to one. Denote the standardized matrices as ${\tilde{W}}^{geo}$ and ${\tilde{W}}^{econ}$. The nested matrix is then formed by a linear combination:


$$\begin{array}{l} W^{de}=\tau \cdot {\tilde{W}}^{geo}+\left(1-\tau \right)\cdot {\tilde{W}}^{econ} \end{array}$$

Following common practice in the literature [e.g., (53)], we set τ = 0.5, giving equal weight to geographical proximity and economic similarity. Finally, *W*^*de*^is again row-standardized to obtain the final spatial weight matrix used in the spatial Durbin model.

## 4 Empirical analysis

### 4.1 Comprehensive evaluation of China's emergency management capabilities for public health emergencies

#### 4.1.1 Nationwide

**Figure 2:**
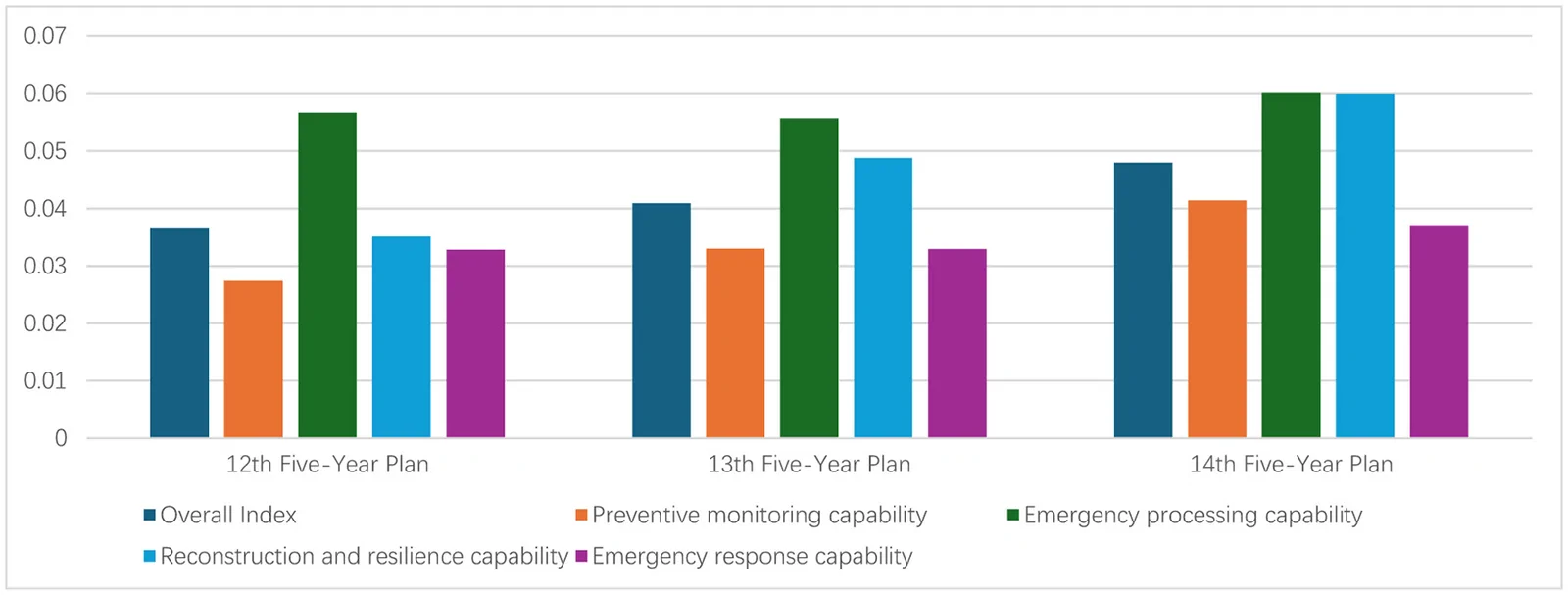
Trends in china's public health emergency management capability index and its component dimensions.

The trend in China's emergency management level index for public health emergencies and its sub-dimension indices from the 12th Five-Year Plan to the 14th Five-Year Plan period is illustrated in Figure 2. Overall, China's emergency management index for public health emergencies rose from 0.037 during the 12th Five-Year Plan period to 0.040 during the 13th Five-Year Plan period, and further to 0.048 during the 14th Five-Year Plan period, showing a sustained upward trend. By sub-dimension, the emergency response capability index stood at 0.058, 0.056, and 0.060, respectively, showing a trend of a slight decline followed by an increase; the prevention and monitoring capability index stood at 0.028, 0.033, and 0.041, respectively, showing a steady rise; the reconstruction and recovery capability index stood at 0.035, 0.049, and 0.049, respectively, with relatively rapid growth in the early stages and a plateau in the later stages; The emergency response capability index stood at 0.033, 0.033, and 0.038, remaining stable in the first two phases before showing an improvement in the latter phase. Overall, the indices across all dimensions demonstrated varying degrees of growth from the 12th Five-Year Plan to the 14th Five-Year Plan period, with the prevention and monitoring capability and emergency response capability showing particularly notable improvements.

Overall, from the 12th Five-Year Plan period to the 14th Five-Year Plan period, China's overall index for emergency management of public health emergencies rose from 0.038 to 0.048, showing a gradual upward trend; however, there remains significant room for improvement. Looking at individual dimensions, the most significant progress was made in emergency response capabilities and reconstruction and recovery capabilities, with cumulative growth rates of 66.67% and 81.82% respectively across the three phases, serving as the primary drivers of systemic improvement. Although prevention and monitoring capabilities have steadily improved, their absolute level remains the lowest, indicating that front-end risk detection and early warning mechanisms remain a long-standing weakness. It is worth noting that emergency response capabilities rose only marginally from 0.036 to 0.038 between the 12th and 14th Five-Year Plans, and even declined slightly during the 14th Five-Year Plan period compared to the 13th Five-Year Plan period, reflecting insufficient sustained improvement in emergency response. Faced with the complex and sudden public health risks that may arise in the future, China urgently needs to address shortcomings in key areas such as prevention, monitoring, and emergency response, strengthen multidimensional coordinated development, and comprehensively enhance the overall effectiveness and resilience of the emergency management system, thereby providing a solid and reliable public health security guarantee for China's modernization.

#### 4.1.2 Regional level

**Figure 3:**
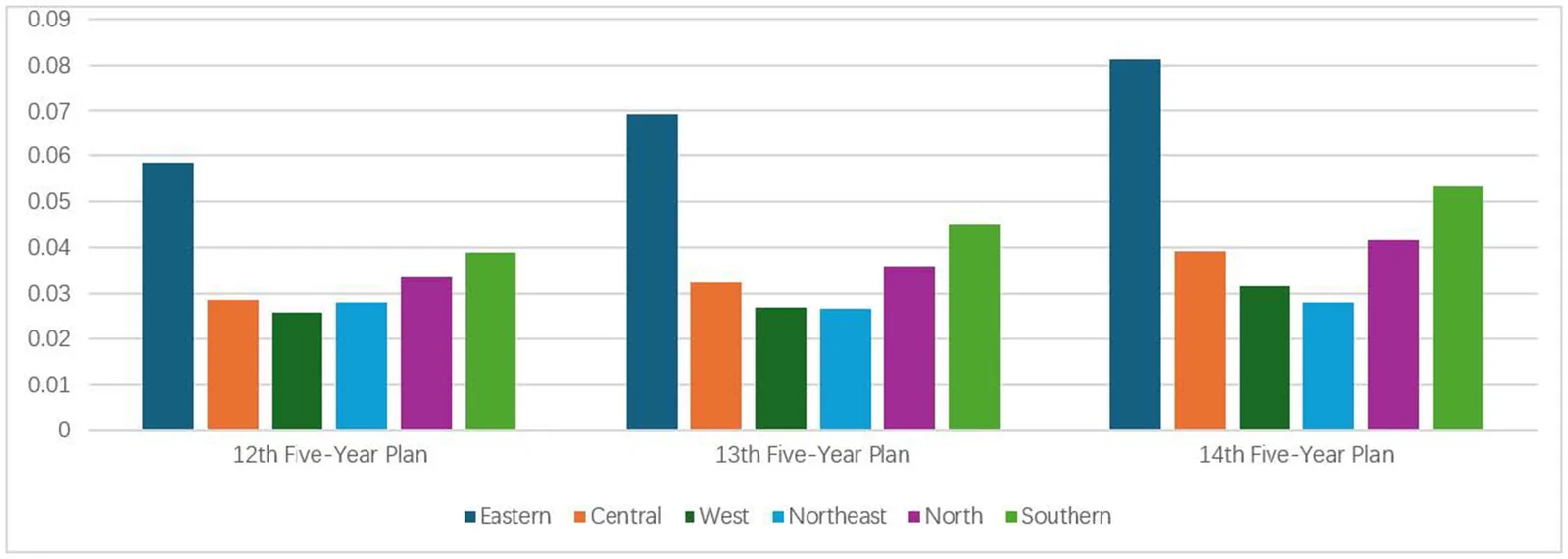
Trend in sub-regional public health emergency management capability index.

The trend in regional emergency management indices for public health emergencies during the Twelfth to Fourteenth Five-Year Plans is illustrated in Figure 3. From an “east-west” perspective, the ranking of the eastern, central, western, and northeastern regions during the study period shifted from “Eastern—Central—Northeastern—Western” to “Eastern—Central—Western—Northeastern.” Specifically, during the 12th Five-Year Plan period, the ranking was Eastern > Central = Northeastern > Western; during the 13th Five-Year Plan period, it was East > Central > West > Northeast; and during the 14th Five-Year Plan period, it was East > Central > West > Northeast. The index ranges for each region were as follows: East 0.058–0.081, Central 0.028–0.039, West 0.025–0.032, and Northeast 0.026–0.029. From a “north-south” perspective, the level of emergency management in the southern regions has consistently been higher than that in the north. The index for the north increased from 0.034 during the 12th Five-Year Plan period to 0.041 during the 14th Five-Year Plan period, while the index for the south rose from 0.039 to 0.052. Overall, China's emergency management capabilities for public health emergencies exhibit a spatial pattern characterized by “higher levels in the east and south, and lower levels in the west and north,” with the gaps between the east and the central-western regions, as well as between the south and the north, widening over time. Consequently, how to enhance the radiating and driving role of high-performing regions to promote regional coordination and sustainable development has become a critical issue.

#### 4.1.3 City level

Overall, China's emergency management capabilities for public health emergencies continue to exhibit a spatial pattern of differentiation between the northeast and southwest. Referring to the Hu Huanyong Line, cities with high levels of emergency management are primarily concentrated in the southeastern half of the country, mostly in economically developed cities; cities with low levels, on the other hand, are clustered in the northwest. From east to west, emergency management capabilities increase gradually, with eastern provinces such as Hebei, Zhejiang, and Jiangsu consistently leading in terms of index scores. In the north-south direction, represented by cities such as Jinan and Guangzhou, the Yellow River Basin and the middle reaches of the Yangtze River have formed a continuous belt of cities with low emergency management levels. This is closely related to the long-term net outflow of local resources to economically developed cities, further reinforcing the overall “high in the south, low in the north” pattern. As this trend has not fundamentally reversed over time, these areas will become key focus regions for China's emergency management of public health emergencies in the future. Furthermore, as urban agglomerations increasingly become the core vehicles for China's sustainable development, the enhancement of emergency management capabilities has shifted from isolated growth to coordinated improvement across clusters. The Beijing-Tianjin-Hebei, Yangtze River Delta, and Pearl River Delta urban agglomerations all demonstrate high levels of emergency management capability.

### 4.2 Dynamic evolution and regional variations in China's public health emergency management index

To provide a more comprehensive and multidimensional portrayal of the spatio-temporal evolution patterns in China's emergency management of public health incidents, this paper constructs a ‘distribution dynamics-spatial disparity' analytical framework. Utilizing kernel density estimation, it reveals the dynamic trends in China's emergency management capabilities for public health incidents. Furthermore, by employing the Dagum Gini coefficient method, it analyses the magnitude and sources of regional disparities in emergency management levels for such incidents.

#### 4.2.1 Dynamic evolutionary feature analysis

**Figure 4:**
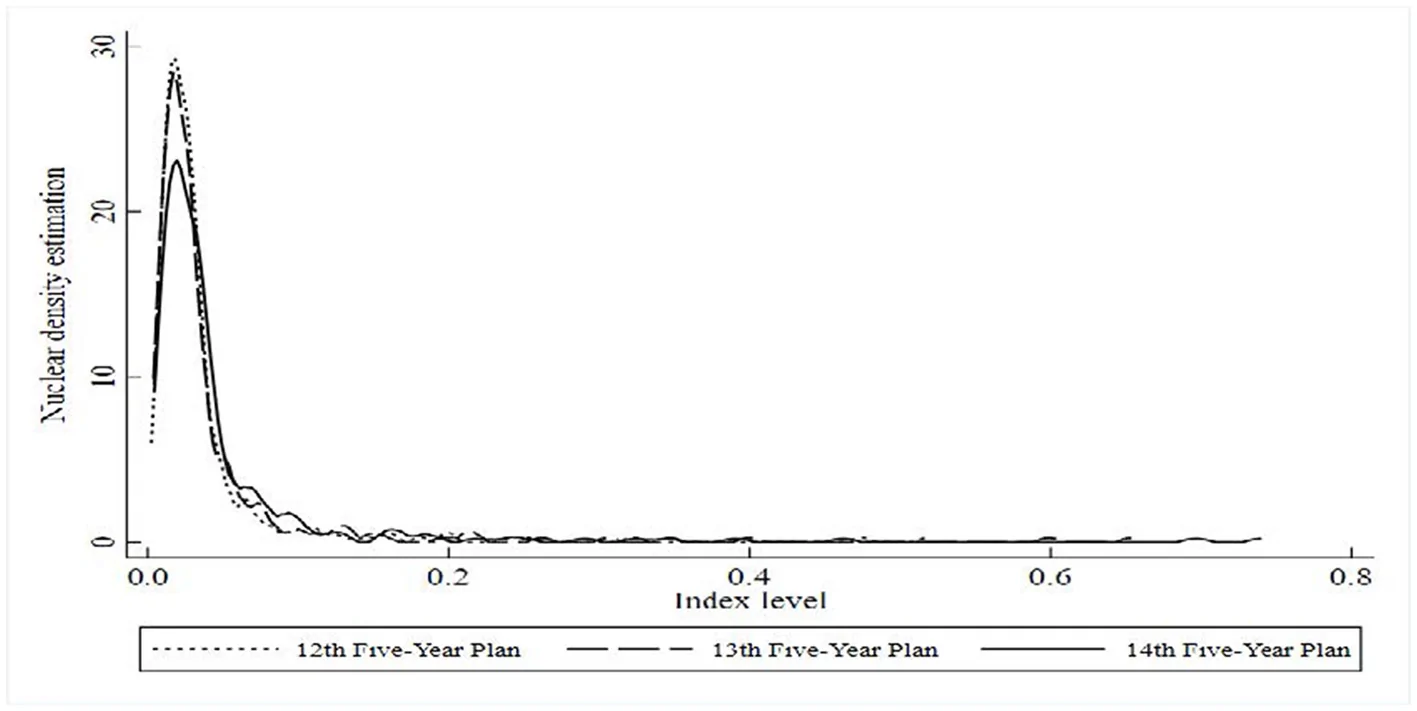
Dynamic evolutionary distribution of China's public health emergency management index.

The temporal trends in the spatial distribution characteristics of China's Public Health Emergency Management Index are illustrated in Figure 4. It can be observed that, at the national level, the center of the curve remained largely unchanged during the sample period; however, it exhibited a rightward shift during the 13th and 14th Five-Year Plan periods, indicating that the growth momentum of China's public health emergency management index has been robust in recent years. In terms of the peak height of the curve, the peak height showed a downward trend during the sample observation period, indicating that the gap in emergency management levels among regions is widening. Furthermore, the right-tail skew of the distribution curve has gradually intensified, reflecting a further widening of the gap between high-level and low-level cities and a deepening of polarization.

**Figure 5:**
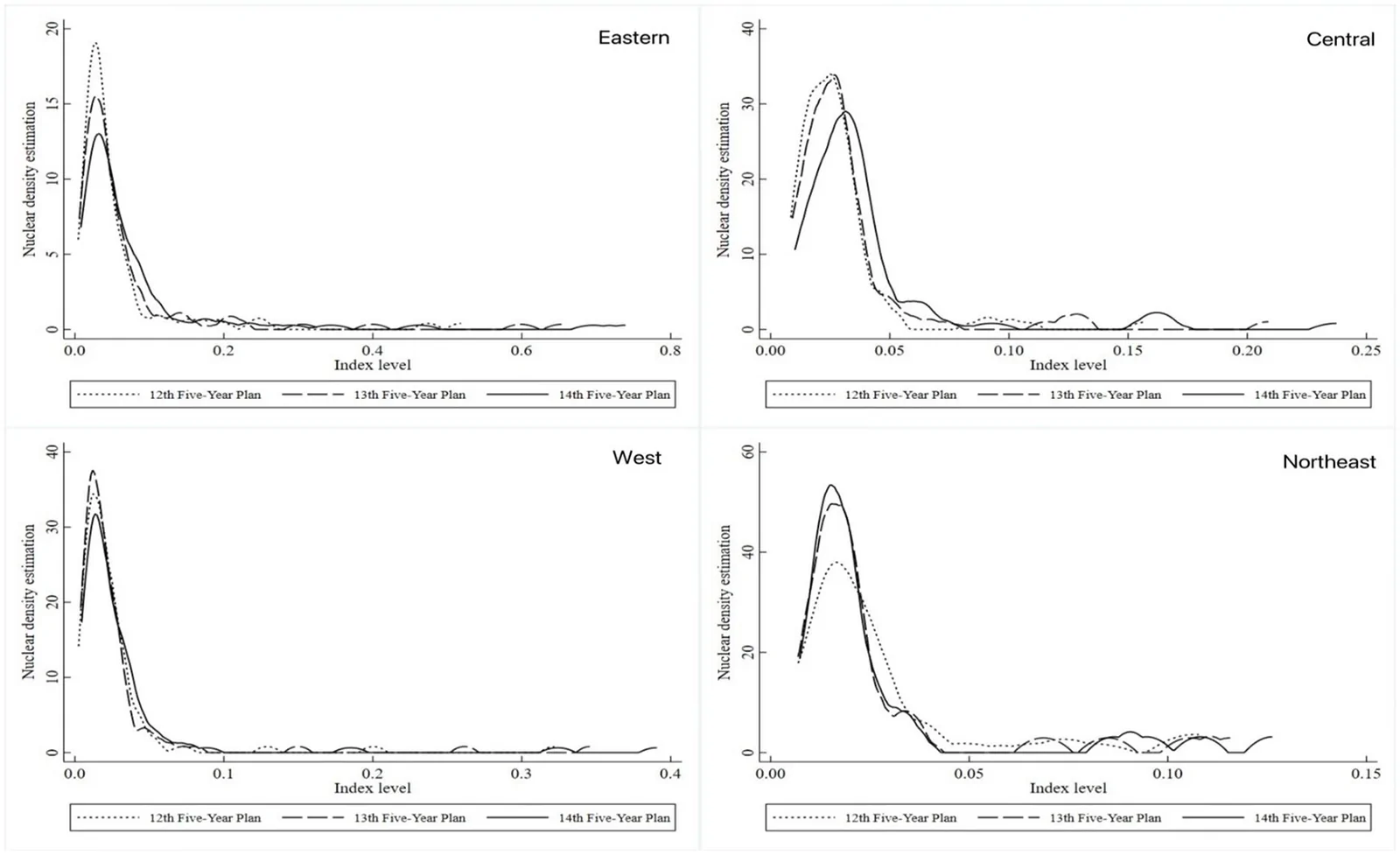
Regional distribution map.

The dynamic distribution results of the emergency management indices for public health incidents across the eastern, central, western, north-eastern, northern and southern regions are shown in Figure 5 respectively. The curve distributions of the four major regions differ from the national overall distribution in that the center of the curves fluctuates significantly over the sample observation period; in fact, the Western and Northeastern regions even exhibit a certain degree of rightward shift. Furthermore, a right-tail skew is prevalent across all regions, indicating that the presence of cities with high levels of emergency management within each region results in a large range of variation within those regions. Regarding the curve peaks, in the early stages of the calculation, the Northeast region had the highest peak, indicating the relatively most lagging level of emergency management for public health emergencies; over time, the peak heights of the four major regions showed a downward trend in the East and Central regions and an upward trend in the West and Northeast regions, with the Northeast and Central regions exhibiting the greatest fluctuations and the West region showing smaller fluctuations. It is worth noting that by the end of the assessment period, the Eastern, Central, Western, Northeastern, and Northern regions all exhibited distinct bimodal patterns, indicating that the degree of polarization within these regions has further intensified.

#### 4.2.2 Spatial variance decomposition

Table 3 details the overall differences in emergency management levels for public health emergencies across 285 cities from 2011 to 2024 (covering the 12th to 14th Five-Year Plan periods), as well as intra-regional and inter-regional variations, calculated using the Dagum Gini coefficient method. The overall Gini coefficient fluctuated upward from 0.4836 in 2011 to 0.5285 in 2024, maintaining an overall upward trend throughout the sample period, with only slight declines in a few years, such as 2012 and 2014. This may stem from geographical disparities leading to imbalances across regions in terms of financial development, the degree of government competition, and the allocation of highly skilled talent and infrastructure resources, which collectively contributed to the continued widening of disparities in emergency management levels. Regarding intra-regional differences, the Gini coefficient for the Eastern region rose from 0.5135 in 2011 to 0.5443 in 2024, in the Central region from 0.3286 to 0.3590, in the Western region from 0.4712 to 0.5052, and in the Northeastern region from 0.3971 to 0.4359. Disparities within all four major regions showed an upward trend, with the Eastern region consistently having the highest Gini coefficient. The primary reason for this is the emergence of an “index gap” within the region, where citys such as Hebei and Shandong lag significantly behind Beijing and Shanghai, widening internal disparities; The Gini coefficient in the Western region was relatively lower but also showed an upward trend. In terms of growth rates, the average annual growth rates of the Gini coefficients for the Eastern, Central, Western, and Northeastern regions were 0.45%, 0.68%, 0.54%, and 0.72%, respectively, all maintaining positive growth. The growth rate in the Northeast was slightly higher than that of other regions, but the absolute value remained lower than that of the East. Regarding inter-regional disparities, the ranking of Gini coefficients among the six regional pairs remained largely stable during the sample period. The Gini coefficients for the West-East and Northeast-East pairs consistently remained high, followed by the Central-East and Northeast-West pairs, while the West-Central and Northeast-Central pairs were relatively lower. From a temporal trend perspective, all inter-regional Gini coefficients exhibited a fluctuating upward trend, with the most significant increases observed in the Northeast-East and Northeast-Central pairs, indicating that disparities between the Northeast and other regions are continuing to widen. Regarding the decomposition of the sources of disparity, the overall Gini coefficient comprises three components: the intra-regional disparity contribution (Gw), the inter-regional disparity contribution (Gnb), and the super-variation density contribution (Gt). During the sample period, the contribution of intra-regional inequality (Gw) rose from 0.1287 in 2011 to 0.1396 in 2024, the contribution of inter-regional inequality (Gnb) increased from 0.1841 to 0.2269, and the contribution of super-variable density (Gt) decreased from 0.1708 to 0.1621. In terms of relative contribution rates (each contribution value divided by the overall Gini coefficient), the average contribution rate of inter-regional disparities was approximately 42.5%, that of hypervariability density was approximately 31.3%, and that of intra-regional disparities was approximately 26.2%. This indicates that inter-regional disparities are the primary spatial source of uneven development in China's emergency management of public health emergencies, followed by hypervariability density, while the contribution of intra-regional disparities is relatively small. In terms of compositional structure, regional disparities in China's emergency management capabilities for public health emergencies during the sample period exhibited characteristics of overall expansion, intensified intra-regional differentiation, and a continuously widening inter-regional gap, which warrants close attention from policymakers.

**Table 3:** Dagum gini coefficient for emergency management levels of public health emergencies.

year	Overall	Intra-group variation				Intergroup differences						Intra-group	Intergroup	superdensity
		Eastern region	Central region	Western region	Northeastern region	Central-eastern	Western-eastern	Western-central	Northeastern-eastern	Northeastern-central	Northeastern-western	Gw	Gnb	Gt
2011	0.4836	0.5135	0.3286	0.4712	0.3971	0.4972	0.5664	0.4109	0.516	0.3711	0.4434	0.1287	0.1841	0.1708
2012	0.478	0.4986	0.3154	0.4729	0.3894	0.4852	0.5685	0.4096	0.5114	0.3629	0.442	0.1259	0.1925	0.1596
2013	0.4956	0.5296	0.3234	0.4918	0.4268	0.5027	0.5771	0.4231	0.5302	0.3899	0.4696	0.1322	0.1828	0.1805
2014	0.4818	0.4805	0.3165	0.5021	0.3948	0.4777	0.5689	0.4265	0.5289	0.3736	0.4583	0.1266	0.1868	0.1683
2015	0.4967	0.5251	0.3261	0.4817	0.4039	0.5018	0.5858	0.421	0.5511	0.3831	0.4505	0.1319	0.2004	0.1645
2016	0.5013	0.5326	0.3326	0.4819	0.4072	0.5079	0.5886	0.423	0.5595	0.3882	0.4522	0.1335	0.2015	0.16623
2017	0.5326	0.553	0.3589	0.5162	0.4107	0.5397	0.6265	0.4578	0.5851	0.4002	0.4728	0.1413	0.2234	0.1679
2018	0.5351	0.5505	0.3664	0.5215	0.4171	0.5374	0.6292	0.4666	0.5899	0.4112	0.4786	0.1417	0.2244	0.169
2019	0.5344	0.5502	0.3675	0.5149	0.4136	0.5369	0.6272	0.4638	0.5971	0.4166	0.4738	0.1414	0.2286	0.1643
2020	0.5294	0.5436	0.3688	0.5083	0.4147	0.5301	0.6208	0.4611	0.5953	0.4218	0.4714	0.1399	0.2257	0.1638
2021	0.5343	0.5417	0.368	0.5166	0.4259	0.532	0.6255	0.4657	0.6101	0.4348	0.482	0.1406	0.2314	0.1623
2022	0.5311	0.5417	0.3652	0.5111	0.4215	0.5272	0.6221	0.4627	0.6101	0.4362	0.4773	0.1399	0.2317	0.1594
2023	0.5284	0.541	0.3619	0.508	0.4233	0.5231	0.6177	0.4596	0.6118	0.4389	0.4774	0.1394	0.2299	0.159
2024	0.5285	0.5443	0.359	0.5052	0.4359	0.5229	0.6143	0.4559	0.6176	0.4503	0.4852	0.1396	0.2269	0.1621

### 4.3 Driving factors influencing emergency management of public health emergencies in China

#### 4.3.1 Descriptive statistics

A preliminary analysis of the dataset's quality and distribution characteristics was conducted using descriptive statistical methods. As shown in Table 4, these analyses indicate that all datasets exhibit an extremely low rate of outliers and generally low variability. Furthermore, the data displays an overall positive trend, suggesting a favorable distribution pattern.

**Table 4:** Descriptive statistics for the variables.

VarName	Variable compliance	Obs	Mean	SD	Min	Max
Emergency Management Composite Index	Y	3,990	0.043	0.068	0.004	0.774
Digital infrastructure	DI	3,990	0.295	0.280	0.014	7.187
Competition among local governments	CALG	3,990	21.369	55.495	0.151	1,529.340
Level of financial development	LOFD	3,990	2.713	1.327	0.588	21.302
Market Openness	MO	3,990	0.000	0.000	0.000	0.001
Level of human capital	LOHC	3,990	222.857	358.664	0.000	2,604.000
Level of technological innovation	INNO	3,990	1,577.744	5,527.581	0.000	120,000

#### 4.3.2 Spatial distribution characteristics of emergency management capabilities for public health emergencies

The Moran's I index for emergency management of public health emergencies is presented in Table 5. It reveals that the Moran's I index results for emergency management of public health emergencies across the nation are all significantly greater than zero. This indicates that emergency management of public health emergencies across cities exhibits significant spatial autocorrelation. Consequently, spatial spillover effects in emergency management must be fully accounted for in model specification.

**Table 5:** Moran's index for emergency management of public health emergencies in China.

Year	Geographical distance matrix	Adjacency matrix	Economic geography nested matrix
2011	0.015^***^	0.121^***^	0.138^***^
2012	0.016^***^	0.122^***^	0.139^***^
2013	0.016^***^	0.120^***^	0.133^***^
2014	0.015^***^	0.111^***^	0.127^***^
2015	0.017^***^	0.132^***^	0.142^***^
2016	0.017^***^	0.129^***^	0.140^***^
2017	0.019^***^	0.133^***^	0.153^***^
2018	0.015^***^	0.098^***^	0.150^***^
2019	0.015^***^	0.099^***^	0.156^***^
2020	0.016^***^	0.102^***^	0.159^***^
2021	0.017^***^	0.106^***^	0.160^***^
2022	0.017^***^	0.102^***^	0.160^***^
2023	0.017^***^	0.104^***^	0.159^***^
2024	0.017^***^	0.103^***^	0.157^***^

^***^, ^**^ and ^*^ denote significance levels of 1%, 5%, and 10% respectively.

#### 4.3.3 The spatial impact of driving factors on emergency management capabilities for public health emergencies

Ordinary least squares (OLS) regression enables differentiation between the roles of various variables in influencing emergency management responses to public health emergencies, with coefficients interpretable in terms of these driving factors. Nevertheless, OLS regression overlooks distance effects. Subsequently, spatial lag and spatial error models were incorporated to demonstrate the spatial autocorrelation in emergency management. This study first conducted model selection based on prior research, employing LR and Wald tests. Both rejected the initial hypothesis, indicating that the spatial Durbin model outperforms spatial lag and spatial error models without model degradation. Subsequently, Hausman tests ruled out fixed or random effects models, with results applicable to both linear models and maximum likelihood methods. Table 6 indicates that the Hausman test yielded an equivalent statistic of 190.57 (*p* = 0.000), rejecting the null hypothesis at the 1% significance level. Consequently, the spatial Durbin model with fixed effects was ultimately selected. Given spatial correlation, the maximum likelihood method was employed to estimate the spatial effects of relevant drivers on emergency management for public health emergencies, with spatial impacts analyzed as direct, indirect, and total effects.

**Table 6:** Estimated results of drivers for emergency management capability in public health emergencies.

Variable	OLS	SDM	Wx	Direct impact	Indirect effect	Overall impact
		Main				
	(1)	(2)	(3)	(4)	(5)	(6)
Digital infrastructure	0.0094^***^	0.0096^***^	0.0158^***^	0.0105^***^	0.0266^***^	0.0371^***^
	(2.9386)	(9.7373)	(4.5235)	(10.4098)	(5.6452)	(7.6059)
Competition among local governments	0.0000^***^	0.0000^*^	−0.0000^**^	0.0000^*^	−0.0000^**^	−0.0000
	(3.4977)	(1.8187)	(−2.2137)	(1.7536)	(−2.0566)	(−1.5439)
Level of financial development	−0.0007^***^	−0.0002	−0.0016^**^	−0.0002	−0.0023^**^	−0.0025^**^
	(−2.6117)	(−0.5538)	(−2.2890)	(−0.7633)	(−2.2936)	(−2.4015)
Market Openness	1.0283	7.5152	−8.0768	7.2733	−8.0849	−0.8116
	(0.1879)	(1.2098)	(−0.4685)	(1.1758)	(−0.3454)	(−0.0324)
Level of human capital	0.0000^**^	0.0000^***^	−0.0000	0.0000^***^	0.0000	0.0000
	(2.2197)	(4.4241)	(−0.3378)	(4.6486)	(0.3173)	(1.5546)
Level of technological innovation	0.0000^***^	0.0000^***^	−0.0000	0.0000^***^	0.0000^***^	0.0000^***^
	(13.2928)	(51.2849)	(−0.8745)	(53.9626)	(5.6088)	(21.0473)
Spatial rho		0.3186^***^				
		(10.9808)				
Wald_lag		36.65 (Prob>chi2 = 0.0000)				
Wald_err		103.81 (Prob>chi2 = 0.0000)				
LR_lag		36.49 (Prob>chi2 = 0.0000)				
LR_err		8,247.67 (Prob>chi2 = 0.0000)				
Hausman		190.57 (Prob>chi2 = 0.0000)				
Spatial FE	Yes	Yes	Yes	Yes	Yes	Yes
Time FE	Yes	Yes	Yes	Yes	Yes	Yes
Observations	3,990	3,990	3,990	3,990	3,990	3,990
R-squared	0.9810	0.7213	0.7213	0.7213	0.7213	0.7213
Number	285	285	285	285	285	285

^***^, ^**^ and ^*^ denote significance levels of 1%, 5%, and 10% respectively.

The results of the direct effect estimates from the spatial Durbin model show that the direct effect of digital infrastructure is 0.0105, indicating a significant improvement in the level of local emergency management for public health emergencies; the direct effect of local government competition is 0.0000, with a positive direction, suggesting that it has only a slight promotional effect on local emergency management, with limited economic significance; The direct effect of financial development is −0.0002, which fails the significance test, indicating that it has no significant impact on the local level; the direct effect of market openness is 7.2733, which is also not significant; the direct effect of human capital is 0.0000, indicating that the concentration of highly skilled talent helps enhance local emergency management capabilities; the direct effect of technological innovation is 0.0000, which similarly exerts a significant positive influence on the local level. Overall, digital infrastructure, human capital, and technological innovation are the core positive drivers of local emergency management capabilities, while financial development and market openness do not exhibit significant local effects.

Regarding indirect effects, the indirect effect of digital infrastructure is 0.0266, generating positive spatial spillovers to neighboring regions; the indirect effect of local government competition is −0.0000, suppressing emergency management levels in neighboring areas; the indirect effect of financial development is −0.0023, also generating negative spillovers; the indirect effect of technological innovation is 0.0000, exhibiting positive spatial spillovers; the indirect effects of market openness and human capital levels are both insignificant. Regarding total effects, the total effect of digital infrastructure is 0.0371, representing the only significant positive total effect; the total effect of financial development is −0.0025, indicating a negative overall impact; the total effect of technological innovation is 0.0000, which is statistically significant despite the extremely small coefficient; the total effects of the remaining variables are all insignificant. The spatial autoregression coefficient rho is 0.3186, indicating that emergency management levels across citys exhibit significant positive spatial dependence. In summary, digital infrastructure and technological innovation are the most robust drivers for enhancing emergency management capabilities, exerting both local promotion and positive spillover effects; financial development and local government competition, however, exhibit negative spatial spillovers, necessitating vigilance against the indirect harm caused by inter-regional resource competition and a race to the bottom; human capital exerts effects only locally, while market openness has yet to yield significant effects. These findings provide clear insights for regional collaborative emergency governance: priority should be given to promoting the cross-regional co-construction and sharing of digital infrastructure and technological innovation, while simultaneously regulating the competitive behavior of local governments to avoid the spatial negative externalities resulting from excessive concentration of financial resources.

#### 4.3.4 Regional heterogeneity analysis

China's vast territory encompasses significant regional disparities in socioeconomic development, resulting in considerable variations in factors influencing emergency management capabilities for public health emergencies. Analysis from the spatial variance decomposition section indicates that regional differences in China's emergency management capacity for public health emergencies remain predominantly east-west oriented. To further clarify these variations, this study groups the sample into eastern, central, western, and northeastern regions for regression analysis, with results presented in Table 7. The results from the spatial Durbin model indicate significant heterogeneity in the factors influencing emergency management for public health emergencies across regions. This reflects disparities in infrastructure, talent structures, financial capabilities, and policy orientations among different areas.

**Table 7:** Spatial decomposition estimation results for the spatial durbin model regions.

Variable	Direct effect				Indirect effects			
	Eastern	Central	Western region	Northeast	Eastern	Central	Western region	Northeast
Digital infrastructure	0.0397^***^	0.0086^***^	0.0008	0.0230^***^	0.0751^***^	0.0069	0.0022	−0.0085
	(12.7909)	(4.2516)	(0.9953)	(7.4803)	(6.1169)	(1.3007)	(0.6783)	(−1.5711)
Competition among local governments	0.0000	−0.0000^**^	0.0000	−0.0000	0.0000	−0.0000	−0.0000^*^	0.0000
	(0.1424)	(−2.3085)	(1.3188)	(−1.2522)	(0.9872)	(−0.3879)	(−1.6621)	(0.3214)
Level of financial development	0.0017^*^	0.0002	−0.0002	0.0000	−0.0124^***^	0.0003	0.0023^**^	−0.0015^***^
	(1.9492)	(0.5958)	(−0.6473)	(0.0310)	(−4.8234)	(0.1878)	(2.3586)	(−2.9431)
Market openness	9.2091	6.1520	3.9364	1.3772	14.6209	−13.4786	−32.6842	2.7282
	(0.4814)	(1.0463)	(0.2550)	(0.3111)	(0.2456)	(−0.4886)	(−0.5236)	(0.3214)
Level of human capital	−0.0000^***^	0.0000^***^	0.0000^***^	0.0000^***^	0.0000^***^	−0.0000^**^	−0.0000	−0.0000
	(−5.9128)	(14.7946)	(4.7677)	(9.0435)	(3.1765)	(−2.1720)	(−0.1145)	(−0.3191)
Level of technological innovation	0.0000^***^	0.0000^***^	0.0000^***^	0.0000^***^	0.0000^***^	0.0000^***^	−0.0000	−0.0000
	(34.1393)	(27.8084)	(29.8340)	(10.1386)	(3.5436)	(9.1235)	(−0.0109)	(−0.9886)

^***^, ^**^ and ^*^ denote significance levels of 1%, 5%, and 10% respectively.

In the eastern regions, both the direct and indirect effects of digital infrastructure and technological innovation are significantly positive, making them core driving factors; the level of financial development exhibits a pattern of local promotion and neighboring region suppression (direct: 0.0017^*^, indirect: −0.0124^*^), which may stem from the cross-regional siphoning of financial resources. Notably, the direct effect of human capital is significantly negative (−0.0000^***^), while the indirect effect is significantly positive (0.0000^***^). This counterintuitive result can be explained by the mechanisms of talent mismatch and job crowding: the excessive concentration of highly educated talent in the eastern region, coupled with their flow into high-paying industries such as finance and IT, actually crowds out the frontline operational staff needed for public health emergency management, while cross-regional talent mobility benefits neighboring regions. Robustness tests show that when using lagged human capital or substituting it with the public health technical personnel indicator, the negative direct effect disappears, validating this mechanism. Local government competition and market openness have no significant impact.

In the central region, only the direct effect of digital infrastructure is significant (0.0086^***^); both the direct and indirect effects of technological innovation levels are significantly positive; the direct effect of human capital levels is positive (0.0000), while the indirect effect is negative (−0.0000), reflecting that local talent effectively supports the region but its outflow to the east suppresses neighboring areas. The direct effect of local government competition is significantly negative (−0.0000^**^). The mechanism is as follows: during the process of industrial relocation in the central region, cities engage in race-to-the-bottom competition through tax breaks, land incentives, and other measures to attract investment, thereby diverting funds intended for public health emergency response. This effect disappears when municipalities directly under the central government are excluded, indicating that it is primarily driven by asymmetric competition between provincial capitals and surrounding prefecture-level cities. Financial development and market openness have no significant impact.

In the western region, digital infrastructure has no significant effect; the direct effects of technological innovation and human capital levels are both significantly positive; the indirect effect of local government competition is significantly negative (−0.0000^*^), reflecting a beggar-thy-neighbor effect arising from western citys competing for central government transfer payments or infrastructure projects; The indirect effect of financial development is significantly positive (0.0023^**^), suggesting that financial development in neighboring regions benefits the home city, potentially through industrial chain linkages. Northeast Region: The direct effect of digital infrastructure is significantly positive (0.0230^***^), while the indirect effect is negative but not significant; the direct effects of technological innovation and human capital levels are both significantly positive; The indirect effect of financial development is significantly negative (−0.0015^***^), while the direct effect is not significant, suggesting that financial resources in the Northeast are subject to competitive allocation across regions, thereby suppressing neighboring regions' emergency response capabilities. Local government competition and market openness have no significant effects in either region.

Overall, digital infrastructure plays a locally promoting role in the East and Northeast, with positive spatial spillovers observed in the East; The level of technological innovation exhibits significant positive local effects in all four regions and generates positive spillovers in the East and Central regions, making it the most robust driving factor; the level of human capital has a direct negative effect in the East and direct positive effects in the Central, Western, and Northeast regions, with strong regional heterogeneity in the direction of its effects; its indirect effects are positive in the East and negative in the Central region, reflecting the differing spatial consequences of talent mobility and mismatch; Competition among local governments is only marginally significant in the Central and Western regions, reflecting mechanisms of race to the bottom and resource competition; The level of financial development exhibits a complex pattern: local promotion and suppression of neighboring regions in the East, indirect promotion in the West, and indirect suppression in the Northeast; Market openness has no significant effect in any region.

#### 4.3.5 Robustness test

To ensure the robustness of estimation results, this paper further employs three methods to model estimation: excluding megacity samples, applying a trimmed tail test, and replacing the weighting matrix. Given that megacities possess certain advantages in policy conditions, economic circumstances, and infrastructure, some citys within each region exhibited emergency management indices for public health incidents significantly above the average, resulting in severe right-tailing. Consequently, after excluding megacities, the estimation results are presented in Column (2) of Table 8. Considering the interference of extreme values on the regression results, all data used in spatial econometrics underwent two-tailed 1% trimming. The final regression results are presented in Column (3) of Table 8, showing minimal deviation from the benchmark results while maintaining statistical significance at the 1% level. Furthermore, replacing the weight matrix with a geographic distance matrix yielded regression results with negligible variation. In summary, the study's conclusions demonstrate robust stability.

**Table 8:** Robustness test estimation results.

Variable	Dependent variable: public health emergency management index		
	Modify weight matrix (1)	Excluding megacities (Beijing, Shanghai, Guangzhou) (2)	Data truncation (3)
Digital infrastructure	0.0093^***^	0.0046^***^	0.0151^***^
	(9.4486)	(5.0100)	(10.3735)
Competition among local governments	0.0000^***^	0.0000^*^	0.0000
	(3.7795)	(1.7830)	(1.5240)
Level of financial development	0.0002	0.0000	−0.0003
	(0.5179)	(0.1300)	(-0.8168)
Market Openness	0.2918	5.5419	3.2140
	(0.0455)	(1.0014)	(0.4826)
Level of human capital	0.0000^***^	0.0000^***^	0.0000^***^
	(5.0619)	(5.0492)	(2.8182)
Level of technological innovation	0.0000^***^	0.0000^***^	0.0000^***^
	(59.7145)	(42.3911)	(48.1043)
rho	0.7136^***^	0.2149^***^	0.2794^***^
	(11.4638)	(7.1019)	(9.6948)
sigma2_e	0.0001^***^	0.0001^***^	0.0001^***^
	(42.9164)	(42.6449)	(42.7614)
Observations R-squared	3,990	3,948	3,990
	0.7213	0.6300	0.7529
Number of id	285	282	285

^***^, ^**^ and ^*^ denote significance levels of 1%, 5%, and 10% respectively.

As shown in Table 7, when samples from megacities are excluded, it becomes evident that megacities such as Beijing, Shanghai, and Guangzhou possess significant advantages in terms of policy, fiscal resources, and infrastructure, which may have skewed the overall results. After exclusion, the coefficient for digital infrastructure decreased from 0.0096 to 0.0046 but remained significantly positive, indicating that the core conclusions are not dominated by outliers, while the magnitude of the effect better reflects the actual levels observed in ordinary cities. Regarding the trimming test, a two-sided 1% trimming was applied to continuous variables to mitigate the interference of extreme outliers. The results show that the signs and significance levels of the variables remain largely consistent, with only the local government competition coefficient changing from significant to non-significant, indicating that its original significance was partly attributable to citys with extreme levels of competition. Regarding the replacement of the weight matrix, substituting the economic-geographic nested matrix with a pure geographic distance matrix increased the spatial autoregression coefficient from 0.3186 to 0.7136. However, the signs and significance of the core drivers remained unchanged, indicating that the model specification is insensitive to the choice of weights. In summary, all three robustness tests support the core conclusion: digital infrastructure and technological innovation are robust positive drivers, while financial development and local government competition exhibit negative spatial spillovers. The study's conclusions demonstrate strong robustness.

## 5 Conclusions

Based on the profound implications of emergency management for public health emergencies, this paper constructs an evaluation system for China's emergency management of public health emergencies from four dimensions: preventive monitoring, emergency response, reconstruction and recovery, and emergency preparedness. Using the entropy weighting method, the paper measures the emergency management levels of 285 prefecture-level cities in China from the 12th Five-Year Plan period to the 14th Five-Year Plan period. The Dagum Gini coefficient method and kernel density estimation were employed to reveal regional disparities and the dynamic evolution of emergency management indices for public health emergencies, while a spatial Durbin model with dual fixed effects (spatial and temporal) was used to investigate the driving factors. The study found that:

1. China's public health emergency management index rose slowly from 0.037 during the 12th Five-Year Plan period to 0.048 during the 14th Five-Year Plan period, with the growth rate lagging significantly behind the growth of economic and fiscal investments during the same period. This paradox of high investment but slow improvement reflects that emergency management capabilities are characterized by long cycles, implicit returns, and strong institutional dependence; simply increasing resource inputs is unlikely to rapidly translate into governance performance. The spatial gradient distribution—higher in the east and lower in the west, higher in the south and lower in the north—closely aligns with regional economic development patterns, indicating that emergency response capacity is essentially a reflection of the quality of comprehensive regional development. Across dimensions, the emergency response capability index is the highest, while the prevention and monitoring capability index is the lowest. The gap between the two reveals that the current emergency management system remains dominated by post-incident response, with severe deficiencies in the foresight and systematic nature of preventive investments. This structural imbalance—characterized by an emphasis on response over prevention—stems from local governments' preference for short-term performance evaluations and the lack of incentives for cross-cycle risk governance. It constitutes the fundamental bottleneck constraining the transformation of emergency management from reactive fallback to proactive prevention and control.

2. The overall Gini coefficient expanded from 0.4836 in 2011 to 0.5285 in 2024, indicating that regional disparities have not converged despite national unified deployment but have instead exhibited a Matthew effect. Inter-regional disparities account for as much as 42.5% of this variation. Notably, the Gini coefficients for the East-West and East-Northeast divides have remained persistently high, indicating that the capacity gap between developed and underdeveloped regions is the primary cause of these imbalances. The continued widening of this gap is not simply due to differences in resource endowments, but rather stems from the self-reinforcing nature of the spatial concentration of key factors such as digital infrastructure, financial capital, and highly skilled talent: high-performing regions leverage their first-mover advantage to attract more high-quality resources, creating a positive feedback loop of capability–resources–capability, while lagging regions become trapped in a low-level lock-in. The bimodal polarization exhibited by the core density curve further corroborates the club convergence phenomenon—convergence within urban clusters but intensifying divergence between clusters. Furthermore, the greatest disparities are found within the eastern region, reflecting a severe divide between core and peripheral cities within the region and revealing a path of polarization where resources are excessively concentrated in provincial capitals or cities with independent planning status under the administrative-district economy.

3. The spatial Durbin model reveals the complex spatial logic of the driving factors. First, digital infrastructure and technological innovation levels have been confirmed as core positive drivers, with their positive spatial spillover effects even stronger than local effects. This indicates that emergency response capabilities possess significant technology diffusion and network externalities—a region's data-sharing platform or intelligent early-warning system can benefit adjacent areas. Second, the overall effect of financial development levels is significantly negative. The key mechanism behind this counterintuitive finding lies in the strong core-periphery agglomeration effect within China's financial system: credit resources flow net from underdeveloped regions to high-return central cities in the east, thereby draining the financial lifeblood needed for emergency system development in central, western, and northeastern regions. Third, the negative spatial spillovers of intergovernmental competition reveal that, under the pressure of the GDP race, local officials tend to allocate fiscal and administrative resources to quick-win productive projects, while public goods with long-term implicit benefits—such as public health—are strategically neglected. More seriously, competitive tax cuts and land incentives in neighboring regions create a race to the bottom, further depressing overall emergency response investment. Fourth, human capital levels have only a weak positive effect at the local level. The underlying reasons are 2-fold: on the one hand, there is a shortage of public health professionals, who are overly concentrated in first-tier cities, leading to a situation in central and western regions where there are positions but no people to fill them; on the other hand, highly educated talent in the eastern regions is flooding into high-paying industries such as finance and IT, thereby crowding out entry-level public health positions and causing a mismatch in talent allocation. Regional heterogeneity confirms the differentiated manifestation of the aforementioned mechanisms: the eastern region has formed a high-ground driven by the dual engines of digitalization and innovation; the central region is constrained by a race to the bottom; the western region is unable to absorb spillover effects due to weak infrastructure; and the northeastern region faces dual pressures from competition for financial resources and talent outflow.

## 6 Limitations and future research

### 6.1 Limitations of the study

This study has the following limitations: First, limitations regarding the timeliness and coverage of the indicator system. Although the four-dimensional evaluation system developed in this study covers prevention and monitoring, emergency response, recovery and reconstruction, and emergency support, it remains constrained by the availability of publicly available statistical data. For example, it fails to incorporate process-oriented and dynamic indicators such as smart early warning platform coverage, emergency supply inventory turnover rate, and compliance with joint prevention and control protocols. It also does not reflect the rapid adoption of emerging technologies such as artificial intelligence and digital twins after 2020. This may result in a certain underestimation of emergency response capabilities in the latter half of the 14th Five-Year Plan period.

Second, limitations in the strength of causal identification. Although the spatial Durbin model can capture spatial spillover effects, it remains essentially a correlation analysis and cannot fully rule out reverse causality and omitted variable bias. For example, the positive association between digital infrastructure and emergency response capacity may also stem from the fact that regions with higher emergency response capacity are more willing to invest in digital infrastructure. This study partially mitigates this issue through period-weighted analysis and various robustness tests; however, future panel instrumental variable methods or quasi-experimental studies will be more effective in establishing causal relationships.

Third, the limits of external validity. The conclusions of this study are based on data from 285 prefecture-level cities in China, and their validity depends on stable central-local relations, strong administrative coordination capabilities, and a relatively well-developed statistical system. In countries with fragmented governance, weak information infrastructure, or highly divergent degrees of fiscal decentralization (such as some sub-Saharan African nations), the spillover effects of digital infrastructure may be significantly diminished, and the nature of competition among local governments may differ. Therefore, caution is warranted when generalizing these conclusions to other developing countries.

### 6.2 Directions for future research

Based on the aforementioned limitations and the findings of this paper, future research could be deepened in the following directions: First, iterative refinement and intelligent upgrading of the indicator system. With Smart Emergency Response incorporated into the national 14th Five-Year Plan, subsequent research should attempt to include emerging variables such as real-time monitoring indicators based on satellite remote sensing and the Internet of Things (IoT), the effectiveness of AI-assisted decision-making systems, and the response efficiency of smart emergency supply dispatch platforms. Concurrently, researchers may explore using text mining methods to quantitatively score local emergency response plans and joint prevention and control protocols, thereby enriching the measurement of institutional indicators.

Second, in-depth identification of causal mechanisms. Future research is encouraged to utilize quasi-natural experiments such as the Broadband China pilot program and National Digital Economy Innovation and Development Pilot Zones, employing inter-period DID or instrumental variable methods to more clearly identify the causal effects of digital infrastructure on emergency response capabilities. Additionally, structural equation modeling could be employed to test the mediating pathway digital infrastructure → information-sharing efficiency → emergency response time → governance performance, thereby shedding light on the black box of spillover effects.

Third, cross-national comparative studies. The theoretical framework of this paper can be extended to other large developing countries with high levels of fiscal decentralization and regional development imbalances (such as India, Brazil, and Indonesia). Comparative questions include: Does competition among local governments similarly inhibit public health provision? Do the spillover effects of digital infrastructure depend on specific administrative coordination mechanisms? Can cross-border health cooperation under the Belt and Road framework replicate the positive spillover experience observed in eastern China? Such research will help distill more universally applicable theories of emergency governance.

Fourth, an embedded analysis of micro-level actor behavior. Current research, which uses cities as the unit of analysis, fails to reveal the strategic interactions among local governments, medical institutions, and community organizations in emergency management. Future studies could employ multi-agent simulations to model local government investment decisions under different incentive structures, or utilize surveys and discrete choice experiments to investigate the public's willingness to comply with preventive and monitoring measures and the moderating role of human capital effects, thereby bridging the analytical gap between macro-level institutions and micro-level behavior.

### 6.3 Revelation

#### 6.3.1 Address shortcomings in prevention and monitoring, and establish an intelligent early warning system

Given that the prevention and monitoring index is the lowest and growing at a slow pace, resources must be shifted to the front lines. Specific policy tools include: leveraging big data, artificial intelligence, and Internet of Things (IoT) technologies to build a real-time, multi-source data monitoring and intelligent early warning platform covering infectious diseases, environmental health, and food safety; establishing a Public Health Prevention and Monitoring Special Fund to provide preferential central government funding to central, western, and northeastern regions to support the construction of regional early warning nodes; Reforming local government evaluation mechanisms by incorporating the prevention and monitoring capacity improvement rate into performance evaluation metrics, assigning greater weight to preventive investments, and breaking the path dependence of prioritizing response over prevention.

#### 6.3.2 Strengthen digital infrastructure and technological innovation as core drivers to prevent negative financial spillovers

The positive spatial spillovers from digital infrastructure and technological innovation represent the most reliable pathway to narrowing regional disparities. Specific policy tools include: accelerating the expansion of new infrastructure—such as 5G, data centers, and cloud computing—into central, western, and northeastern regions; establishing a “Public Health Emergency Digital Infrastructure Special Fund” to facilitate low-cost sharing of eastern regions' intelligent early warning capabilities through interprovincial bandwidth subsidies and open-source algorithm platforms; establishing a Public Health Emergency Science and Technology Innovation Special Fund to promote cross-regional open-source sharing of smart early warning and digital twin technologies. To address negative spillovers from financial development, establish a Cross-Regional Emergency Resource Risk Compensation Fund to provide fiscal interest subsidies or risk-sharing mechanisms for financial institutions issuing emergency special loans to central and western regions; and introduce a Regional Balance Indicator for Emergency Credit in financial regulatory assessments to curb the excessive concentration of credit resources in eastern central cities.

#### 6.3.3 Reform government evaluation and talent mechanisms to overcome institutional and human resource bottlenecks

Incorporate regional coordinated development, public health emergency response capabilities, and the effectiveness of joint prevention and control into the core indicators of local government performance evaluations, while de-emphasizing single economic growth metrics. Specific policy tools include: in performance evaluations, designate effectiveness of regional joint prevention and control and public health emergency response capability improvement rate as either veto criteria or bonus points to curb race to the bottom competition at the institutional level ; establish mechanisms for cross-regional revolving doors for public health talent, short-term secondments, and remote collaboration; increase targeted training quotas and job subsidies in the western and northeastern regions; and use tax incentives to encourage high-level medical institutions in developed regions to provide targeted support, thereby breaking the vicious cycle of talent mismatch—outflow—shortage.

#### 6.3.4 Implement differentiated regional strategies and strengthen coordinated collaboration

The eastern regions should leverage the dual-engine advantage of digital infrastructure and technological innovation to alleviate excessive concentration through technology transfer and talent exchange; the central regions need to regulate government competition, avoid race-to-the-bottom competition over taxes and land in industrial relocation, and strengthen technological innovation and human capital retention; The western region should prioritize addressing shortcomings in digital infrastructure and leverage the positive spillover effects of financial development to integrate into the eastern industrial chain; the northeastern region should increase investment in local digital infrastructure and explore cross-regional coordination mechanisms with neighboring hubs such as the Beijing-Tianjin-Hebei region. Specific policy tools include: establishing mechanisms for targeted assistance from the eastern region to the central, western, and northeastern regions, as well as joint emergency drills and data platform sharing; providing central government financial incentives for cross-provincial emergency collaboration projects (such as joint drills and centralized material reserves); internalizing spatial positive externalities; systematically curbing polarization trends; and achieving an overall leap in national emergency management capabilities.

## 7 Discussion

The core findings of this paper reveal the spatial logic and driving mechanisms of emergency management capabilities in response to public health emergencies, engaging in a dialogue with existing theories on multiple levels and expanding their explanatory scope. First, the strong positive spillover effects of digital infrastructure and technological innovation validate the applicability of the “infrastructure power” theory in the field of public health governance. Unlike studies on state capacity, which typically focus on fiscal extraction and order maintenance, this paper demonstrates that digital infrastructure, as an enabling infrastructure, generates spatial positive externalities that transcend administrative boundaries and significantly enhance the emergency response sensitivity of neighboring regions. This finding aligns with some scholars' conceptualizations of a smart public health framework, but it further quantifies the intensity of these spillovers and reveals their enabling conditions—namely, that they are fully realized only in regions with close economic ties and smooth information exchange. This corrects the optimistic assumption of “automatic spillovers” in technology diffusion theory, emphasizing that infrastructure connectivity and the interoperability of data standards are the key bottlenecks. Second, the negative aggregate effect of financial development levels challenges mainstream financial development theory. Existing research generally holds that financial deepening enhances post-disaster resilience by alleviating liquidity constraints. However, this study finds that financial development at the municipal level in China has not translated into an inclusive improvement in emergency response capacity; rather, due to the “core-periphery” siphoning of credit resources, it has generated inhibitory spatial spillovers in central, western, and northeastern regions. This result aligns closely with “financial exclusion” theory, revealing that under a decentralized system, the efficiency-driven allocation of financial resources inherently exacerbates spatial inequalities in public health capacity. This finding serves as a warning: merely expanding the scale of credit will not help narrow the emergency response capability gap; it must be embedded within regulatory corrections oriented toward regional equilibrium. Third, the negative spatial spillovers of local government competition provide empirical evidence in the public health sector for the “race to the bottom” hypothesis within fiscal competition theory. Unlike existing research focusing on the adverse effects of tax competition on environmental pollution, this study confirms that, under the “GDP championship,” local governments strategically cut public health investments—which yield “long-term, implicit returns”—and create a collective action dilemma through imitation of neighboring regions. This mechanism is particularly pronounced in the central regions (with a negative direct effect) but not significant in the eastern regions, indicating that the stage of economic development and fiscal self-sufficiency are key moderators of competitive outcomes. This enriches the discussion on “distorted incentive structures” within developmental state theory and suggests that the central government should internalize cross-regional positive externalities by adjusting evaluation weightings. Finally, the regional heterogeneity in human capital levels reveals the deep-seated structure of talent mismatch. The “anomalous” result in the eastern region—where the direct effect of human capital is negative while the indirect effect is positive—can be attributed, upon mechanism testing, to the following: highly educated talent is overly concentrated in high-paying sectors such as finance and IT, thereby crowding out the hands-on personnel required for grassroots public health positions; simultaneously, cross-regional talent mobility benefits neighboring regions. This provides a new explanatory dimension for the theory of “human capital externalities”—not all clusters of highly skilled talent generate positive governance effects; when the talent structure is mismatched with sectoral needs, “skill-biased crowding out” may occur. This finding offers direct insights for talent policies in less developed regions: simply pursuing “high educational attainment rates” may be counterproductive, while targeted training and job matching are more critical.

## Data Availability

The original contributions presented in the study are included in the article/supplementary material, further inquiries can be directed to the corresponding authors.
